# Health-related quality of life in adolescents with persistent pain and the mediating role of self-efficacy: a cross-sectional study

**DOI:** 10.1186/s12955-020-1273-z

**Published:** 2020-01-30

**Authors:** Erik Grasaas, Sølvi Helseth, Liv Fegran, Jennifer Stinson, Milada Småstuen, Kristin Haraldstad

**Affiliations:** 1 Department of Health and Nursing Science, Faculty of Health and Sport Sciences, University of Agder, Postbox 422, 4604 Kristiansand, Norway; 2 Department of Nursing and Health Promotion, Faculty of Health Sciences, Oslo Metropolitan University, Oslo, Norway; 3 Child Health Evaluative Sciences, The Hospital for Sick Children, Toronto, Canada; 4 Lawrence S. Bloomberg Faculty of Nursing, University of Toronto, Toronto, Canada; 5 Institute for Health Policy, Management, and Evaluation, University of Toronto, Toronto, Canada

**Keywords:** Adolescents, Health-related quality of life, Persistent pain, Self-efficacy, Mediation

## Abstract

**Background:**

Persistent pain has a high prevalence among adolescents. Pain has been shown to reduce all aspects of the adolescent’s health-related quality of life (HRQOL). In adult patients with pain, self-efficacy has been shown to mediate the relationship between pain intensity, disability and depression. However, little is known about whether self-efficacy acts as a mediating variable in the relationship between persistent pain and HRQOL sub-scale scores in a school-based population of adolescents.

**Objectives:**

To describe the experience of pain, HRQOL and self-efficacy, and to explore the association between pain intensity, general self-efficacy and HRQOL in adolescents with persistent pain by testing self-efficacy as a possible mediator.

**Methods:**

The study participants were 78 adolescents with persistent pain, aged 16–19 years, who were recruited from five high schools in southern Norway. All participants completed an electronic survey consisting of the Lubeck Pain Questionnaire, which included a visual analogue scale (VAS) measuring pain intensity, the General Self-Efficacy Scale (GSE) and the KIDSCREEN-52 Questionnaire measuring HRQOL. Statistical analyses were conducted using the PROCESS macro for SPSS developed by Andrew Hayes.

**Results:**

All participants reported pain in multiple locations, of which the head was most common (88.5%). Mean (SD) pain intensity score of the participants was 5.4 (1.8). The study sample had poor HRQOL, with mean (SD) scores for several sub-scales ranging from 45.2 (21.0) to 91.0 (13.3) on a 0–100 scale. The associations between pain intensity and the HRQOL sub-scales of physical well-being, psychological well-being, mood, self-perception, autonomy and school environment were mediated by self-efficacy. The highest degree of mediation and, thus, the largest indirect effect was estimated for the HRQOL sub-scale physical well-being (67.2%).

**Conclusions:**

This school-based sample of adolescents with persistent pain had impaired HRQOL. Up to 67% of the reduction in the HRQOL sub-scale scores for physical well-being, psychological well-being, mood, self-perception, autonomy and school environment could be explained by the mediating variable self-efficacy. Thus, future pain-management interventions that aim to increase HRQOL in school-based populations of adolescents with persistent pain should consider promoting self-efficacy and providing more targeted interventions.

**Trial registration:**

ClinicalTrials.gov ID NCT03551977.

## Introduction

Persistent or chronic pain among adolescents is recognized as a global growing health problem. Headache, abdominal pain and back pain are most commonly reported, but these frequently coexist with persistent pain at multiple locations [1, 2]. Pain in adolescence is often complex, may have no clear cause and can include cycles of flares [3]. Chronic pain is defined as persistent or recurrent pain lasting more than 3 months [4]. Internationally comparable data indicate that persistent pain is highly prevalent among adolescents [5]. Research indicates that the prevalence of persistent pain among adolescents in Western countries ranges from 20 to 35%, is clearly higher in girls than in boys and increases with age [6–11]. The national annual Young-data surveys have revealed an increase in psychosocial complaints among Norwegian adolescents attending high schools, herein about half of the adolescents have concerns like “everything feels like a struggle” [12]. Further, Norwegian adolescents have reported that the feeling of stress and struggle may be a contributing factor to their pain experience [13]. However, persistent pain in a school-based (non-clinical) population of adolescents usually has an unconfirmed aetiology with no underlying pathological condition or apparent single explanation [14]. Thus, further insight into the complexity of pain associations in adolescence is needed.

Persistent pain in adolescence has several consequences. Short-term consequences may include absence from school and social activities, resulting in periods of isolation from peers and role loss, which may explain why adolescents with pain tend to have fewer friends compared with healthy adolescents [3, 15, 16]. In addition, pain that begins in adolescence may have long-term consequences if the adolescents enter adulthood suffering persistent pain, which carries risks of psychosocial and socio-economic distress [17, 18] Other long-term consequences include higher levels of perceived stress, sleep disturbance, reduced physical activity and overall reduced health-related quality of life (HRQOL), which all negatively affect different aspects of the adolescent’s everyday life [19, 20].

HRQOL is a multidimensional concept that includes physical, psychological, social and spiritual aspects of life [21]. The concept of HRQOL is often used when assessing how pain can influence the daily life of adolescents, because pain impacts all aspects of life [22, 23]. Several studies that examined the association between pain and HRQOL among adolescents showed that persistent pain is associated with reduced HRQOL [10, 20, 22–25]. There are several questionnaires that measure HRQOL, of which KIDSCREEN-52 has been shown to have the best structural validity [26]. However, there is limited research investigating HRQOL and pain in a school-based population of adolescents using the 10 sub-scales of the KIDSCREEN-52 questionnaire [27]. A Norwegian study showed that pain in children and adolescents was associated with lower HRQOL demonstrated by reduced scores for all 10 sub-scales of the KIDSCREEN-52 questionnaire, but had the greatest effect on the HRQOL sub-scales of self-perception, psychological well-being, mood, relationship with parents and school environment [27]. Further research on pain and HRQOL in a school-based sample of adolescents is needed to explore whether this association can be explained by underlying mechanisms or is related purely to the pain itself.

Self-efficacy, defined by Albert Bandura as “one’s beliefs in one’s capability to organize and execute the courses of action required to achieve given results”, is well-known to affect a person’s cognition [28, 29]. In adults, general self-efficacy (GSE) has been shown to positively impact QOL by reducing stress and, thereby, increasing QOL [30, 31]. In young adolescents, a higher degree of self-efficacy has been shown to be related to higher HRQOL scores [32], and has been associated with several positive health outcomes for adolescents with chronic pain, including higher self-esteem and acceptance, and lower disability and somatic symptoms [33, 34]. In a sample of adolescents with chronic headache, higher self-efficacy was associated with improved school performance and lower disability [35].

Previous research evidence has shown that self-efficacy acts as an underlying mechanism by mediating the relationship between pain-related fear and school-related disability in adolescents with chronic headache [36]. In adults with chronic pain, self-efficacy was found to be a mediator of the relationship between pain intensity, disability and depression [37]. Bandura has proposed that self-efficacy might act as a mediator between stressful experiences and outcomes such as well-being [38]. However, no study has investigated whether self-efficacy acts as a possible mediator of the relationship between pain and HRQOL in a school-based sample of adolescents.

Thus, the purpose of this study was to describe the pain experience (intensity, frequency, duration and location), HRQOL and GSE in a sample from a school-based population of adolescents with persistent pain, and to assess possible associations between pain intensity, GSE and HRQOL. We hypothesized that pain intensity is negatively associated with HRQOL, and that self-efficacy plays a role as a mediator.

## Methods

### Design and aim

Data for this cross-sectional study were collected at baseline during an intervention study that aimed to help reduce pain and promote HRQOL in Norwegian adolescents with persistent pain using a smartphone application called iCanCope with Pain™.

### Setting of the study

The study was conducted in southern Norway in 2018. All government-funded high schools within an area of 10 miles, were invited to participate. The area includes about 100,000 habitants. No high schools were excluded or disagreed to participate. The parents of the attending adolescents had varied level of education, here used as a proxy for socio-economic status, thus we consider our sample to be representative of a population of adolescents with different levels of socioeconomic status (SES). We included 16–19-year-old adolescents with persistent pain (weekly pain lasting 3 months or more) who were able to read and understand Norwegian and used their own smartphones. Adolescents with cognitive disabilities were excluded because of their inability to understand how to use the *iCanCope with Pain* application, goal setting and/or library readings. Adolescents with pain of pathological or medical origin (e.g., arthritis/oncology patients) were excluded because the program was not specifically designed for these patient groups.

### Procedure

The primary author visited all high schools and informed each class about the study. To ensure anonymity and confidentiality, adolescents received oral and written information in the classroom with an attached email address generated solely for the purpose of this study. Information was also available on the high schools’ websites. Those who wanted to participate in the study could send an email to the research study email address. The data collection period lasted 3 months. All participation was voluntary, and participants provided written informed consent before participating in the study. They were aware that they could withdraw without a reason at any time during the study, in which case their data would be deleted and destroyed, and that the confidentiality and anonymity of their data were ensured at all times. The study was approved by the Norwegian Regional Committee for Medical Research Ethics South-East-B (REK reference 2017/350).

### Measures

The electronic survey tool used in our study was designed to consecutively administer the following respective questionnaires. The adolescents were free to end the electronic survey at any time. Most questions included a neutral option, thus resulting in all items being answered. The electronic survey was pre-tested [39]. The first page of the survey contained demographics information such as age, gender and parental education. Parental education levels were used to indicate the participants’ socioeconomic status (SES).

#### Pain

To assess pain, the Norwegian version of the Lübeck Pain-Screening Questionnaire (LPQ) was administered, which has demonstrated satisfactory content validity and high internal consistency (Cronbach’s alfa 0.92) [6]. The LPQ aims to identify both the presence and consequences of pain with a recall period of 3 months. For the present study, pain intensity was digitally measured using a visual analog scale (VAS) ranging from 0 (no pain) to 10 (worst pain imaginable). This VAS is a well-known measure of pain intensity, has been found to be both valid and reliable [40, 41], and has been validated for digital use [42]. Pain duration was recorded in three categories: pain lasting more than 3 months, more than 6 months or more than 12 months. Pain frequency was defined as how often pain was experienced and was categorized as daily pain, several times a week or once a week. Pain location referred to pain in specific body regions. Multi-site pain was defined as pain in a least two of the following predefined regions used by the LPQ: head, ears, teeth, throat, chest, back, stomach, reproductive organs (pain during menstruation), arms, legs or other locations.

#### HRQOL

To assess HRQOL, the Norwegian-translated and validated version of KIDSCREEN-52 was administered [16]. The KIDSCREEN-52 questionnaire is a cross-cultural multi-dimensional instrument that has been validated in several countries with internal consistency above 0.80 (Cronbach’s alfa) for all dimensions [16, 43, 44], and consists of 52 questions using a 1–5 Likert scale grouped into 10 sub-scales comprised of different numbers of items: physical well-being (five items), psychological well-being (six items), moods and emotions (seven items), self-perception (five items), autonomy (five items), relationship with parents (six items), social support (six items), school environment (six items), bullying (three items) and financial resources (three items) [45]. Next, we followed the KIDSCREEN manual and transformed negative questions into positives [43], after which the data were transformed to a linear 0–100-point scale, where the lowest possible HRQOL scored 0 and the highest HRQOL scored 100.

#### Self-efficacy

To assess self-efficacy, the Norwegian 5-item version of the General Perceived Self-Efficacy Scale (GSE) revised and translated by Røysamb and colleagues (1998) was administered [46]. The GSE scale originally included 10 items and was developed by Jerusalem and Schwarzer [47]. The short form of the GSE scale has also been found to be valid and reliable with satisfactory internal consistency (Cronbach’s alfa 0.82) [48, 49]. GSE is a psychometric scale developed to identify a person’s optimistic self-belief in coping, often defined as one’s global confidence in one’s ability across a wide range of demanding and novel situations [47]. In the independent versions of GSE, all items use a 1–4-point scale, where 1 refers to the lowest GSE and 4 the highest. Hence, the total score for the five GSE items ranges from 5 (lowest) to 20 (highest total score), where higher scores indicate higher GSE.

### Statistical analyses

The statistical analyses were conducted using IBM SPSS Statistics for Windows (version 25.0; IBM Corp., Armonk, NY). Demographic data were described using descriptive measures. The study variables pain intensity, GSE and 9 out of 10 HRQOL sub-scales had skewness values of ±0.5 and kurtosis values of ±1, which indicated that these variables are approximately normally distributed. Continuous variables were described by mean and standard deviation, and categorical variables by frequency and percentage. Mediation analysis was conducted using the PROCESS macro bootstrapping method developed for SPSS by Hayes [50], herein we entered SES as a covariate. The mediation effect was regarded as statistically significant if the 95% confidence interval (CI) for this effect did not include zero. Further, a linear regression of the mediator (self-efficacy) on pain was conducted. A correlation matrix between self-efficacy and HRQOL subscales was constructed using Pearson correlations. Finally, we conducted linear regression of HRQOL on both self-efficacy (indirect path) and pain (direct path). The indirect and direct effects were separately divided by the total effect and multiplied by 100 to be presented as a percentage. *P*-values < 0.05 were considered significant and all tests were two-sided. According to Preacher and Hayes, a significant indirect effect does no longer impose evidence of a simple association between the dependent and independent variable as a precondition for a mediation analysis [51]. Hence, all HRQOL sub-scales were included.

**Fig. 1:**
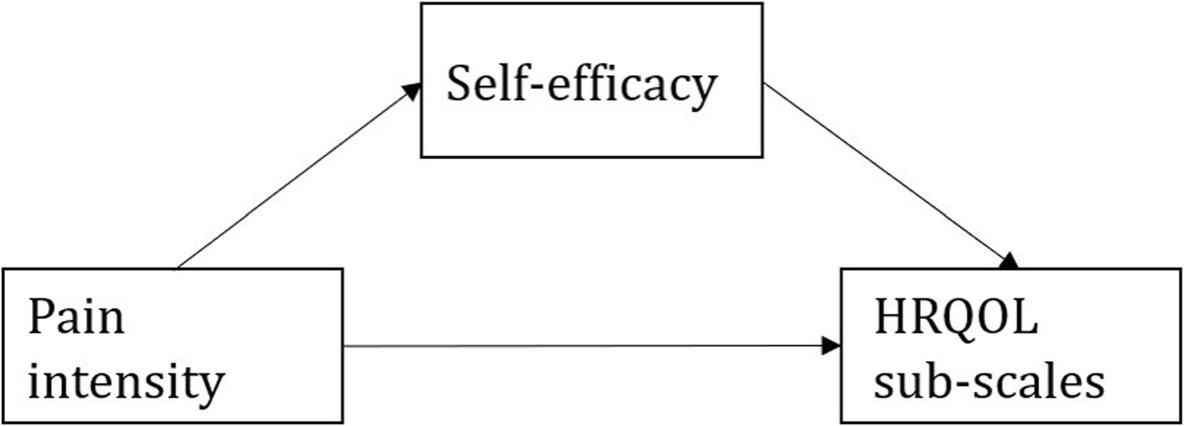
Schematic of our final mediation model

We proceeded using the mediation model depicted in Fig. 1.

## Results

### Participants

About 4000 adolescents from a school-based population were approached to participate, and based on the previous evidence of the prevalence of persistent pain [2, 6–8, 10, 11], we predicted that about one quarter of the approached adolescents would be eligible. One hundred and seventeen adolescents registered for the study by sending an email to the study email address, of whom 83 provided informed consent and completed the baseline questionnaires. We do not have any data for the 34 adolescents who did not continue after registration. Five adolescents were excluded because they did not meet the inclusion criteria (i.e., pain presence). In total, 78 adolescents with persistent pain participated in the study. The majority (62, 79.5%) were girls and 16 (20.5%) were boys. The participants were aged 16 (26.9%), 17 (29.5%), 18 (26.9%) or 19 (16.7%) years old.

### Descriptive data for study variables: pain intensity, HRQOL and GSE

Mean (SD) pain intensity (VAS) score in the study sample was 5.4 (1.8) (Table 1). Girls reported higher mean (SD) pain intensity scores than boys (5.7 [1.8] versus 4.2 [1.9], respectively). The participants’ mean (SD) scores ranged from 45.2 (21.0) to 91.0 (13.3) on a 0–100 scale for the HRQOL sub-scales. Boys reported higher scores than girls for all HRQOL sub-scales except financial resources (see Table 1). The largest gender difference was shown for the HRQOL sub-scale mood, where girls reported a mean (SD) score of 54.9 (21.3) compared with 73.7 (15.6) for boys. The participants reported a mean (SD) GSE score of 13.5 (3.3), with girls scoring 13.2 (3.3) and boys 14.8 (3.2).

**Table 1:** Characteristics of the participants: pain, self-efficacy and HRQOL sub-scale scores

Study variable	All (*n* = 78) mean (SD)	Girls (*n* = 62) mean (SD)	Boys (*n* = 16) mean (SD)
Pain intensity	5.42 (1.88)	5.74 (1.75)	4.19 (1.90)
Self-efficacy	13.54 (3.30)	13.21 (3.29)	14.81 (3.17)
KIDSCREEN subscale			
Physical well-being	45.19 (20.99)	41.37 (18.56)	60.00 (23.80)
Psychological well-being	56.09 (22.23)	53.02 (21.93)	67.97 (19.76)
Mood	58.74 (21.56)	54.90 (21.29)	73.66 (15.64)
Self-perception	45.71 (23.16)	43.06 (22.96)	55.94 (21.62)
Autonomy	59.23 (18.90)	55.73 (18.64)	72.81 (13.16)
Relationship with parents	65.01 (24.41)	64.38 (24.80)	67.45 (23.43)
Financial resources	70.61 (26.85)	71.24 (27.23)	68.23 (26.03)
Social support	60.52 (20.60)	58.67 (20.83)	67.71 (18.54)
School environment	54.75 (20.03)	52.02 (18.32)	65.36 (23.31)
Bullying	91.03 (13.34)	90.99 (14.02)	91.15 (10.74)

### Pain duration, frequency and location

The participants were all affected by the location of pain, and all participants reported multi-site pain during the 3 months recall period (details in Table 2). Almost half of the participants (48.7%) reported pain lasting more than 12 months, with 29.5% reporting daily pain and 46.2% experiencing pain several times a week. More than half of the participants (51.3%) reported pain at locations other than the 10 pre-defined locations; in this unspecified category, pain in shoulder(s), neck and hip was most frequently reported. Headache was most commonly reported by the participants (88.5%), herein 95.2% of the girls and 62.5% of the boys reported headache (Table 2).

**Table 2:** Counts and percentage of bodily regions affected by pain within the 3-month recall period for all participants and stratified by gender

Pain region	All (*n* = 78)	Girls (*n* = 62)	Boys (*n* = 16)
Head	69 (88.5%)	59 (95.2%)	10 (62.5%)
Teeth	15 (19.2%)	14 (22.6%)	1 (6.3%)
Ears	14 (17.9%)	14 (22.6%)	0
Throat	35 (44.9%)	32 (51.6%)	3 (18.8%)
Back	49 (62.8%)	40 (64.5%)	9 (56.3%)
Chest	21 (26.9%)	18 (29.0%)	3 (18.8%)
Stomach	50 (64.1%)	45 (72.6%)	5 (31.3%)
Reproductive organs	50 (64.1%)	50 (80.6%)	0
Arms	12 (15.4%)	8 (12.9%)	4 (25.0%)
Legs	30 (38.5%)	27 (43.5%)	3 (18.8%)
Other	40 (51.3%)	33 (53.2%)	7 (43.8%)

### Associations between pain intensity, HRQOL sub-scale scores and GSE

Scores for all the HRQOL sub-scales and GSE were negatively associated with pain intensity. Pain intensity was a significant predictor of the scores for the HRQOL sub-scales physical well-being (B = −2.81), psychological well-being (B = − 4.55), mood (B = − 3.62), self-perception (B = − 4.13), social support by peers (B = − 3.26) and school environment (B = − 3.18) (Table 3).

**Table 3:** Linear regressions of pain intensity (independent) on HRQOL sub-scales (dependent) and on GSE (dependent)

Variable	B	95% CI	*P* value
Physical well-being	−2.81	−5.27 to −0.34	0.02
Psychological well-being	−4.55	−7.04 to − 2.0.6	< 0.01
Mood	− 3.62	− 6.10 to − 1.14	< 0.01
Self-perception	−4.13	−6.78 to − 1.49	< 0.01
Autonomy	−1.74	− 4.00 to 0.52	0.12
Relationship with parents	−2.47	−5.39 to 0.44	0.09
Financial resources	−1.06	−4.31 to 2.20	0.52
Social support	−3.18	−5.56 to −0.79	0.01
School environment	−3.26	−5.57 to − 0.95	< 0.01
Bullying	−0.87	−2.48 to 0.74	0.29
GSE	−0.63	−1.01 to −2.56	< 0.01

*CI* confidence interval

We examined the association between self-efficacy (mediator) and HRQOL sub-scale scores (dependent variables), which revealed a non-significant relationship between self-efficacy and the HRQOL sub-scale social support. Estimates of the correlation matrix between HRQOL sub-scales and self-efficacy are listed in Table 4 and revealed an overall low to moderate correlations. The strongest correlation was found between HRQOL sub-scale physical well-being and self-efficacy of 0.538.

**Table 4:** Estimates of the correlation matrix between HRQOL and self-efficacy

HRQOL sub-scales	Self-efficacy
Physical well-being	0.538
Psychological well-being	0.414
Mood	0.407
Self-perception	0.490
Autonomy	0.269
Relationship with parents	0.184
Financial resources	0.048
Social support	0.208
School environment	0.327
Bullying	0.010

### Mediation of self-efficacy on the relationship between pain intensity and selected HRQOL sub-scale scores

**Fig. 2:**
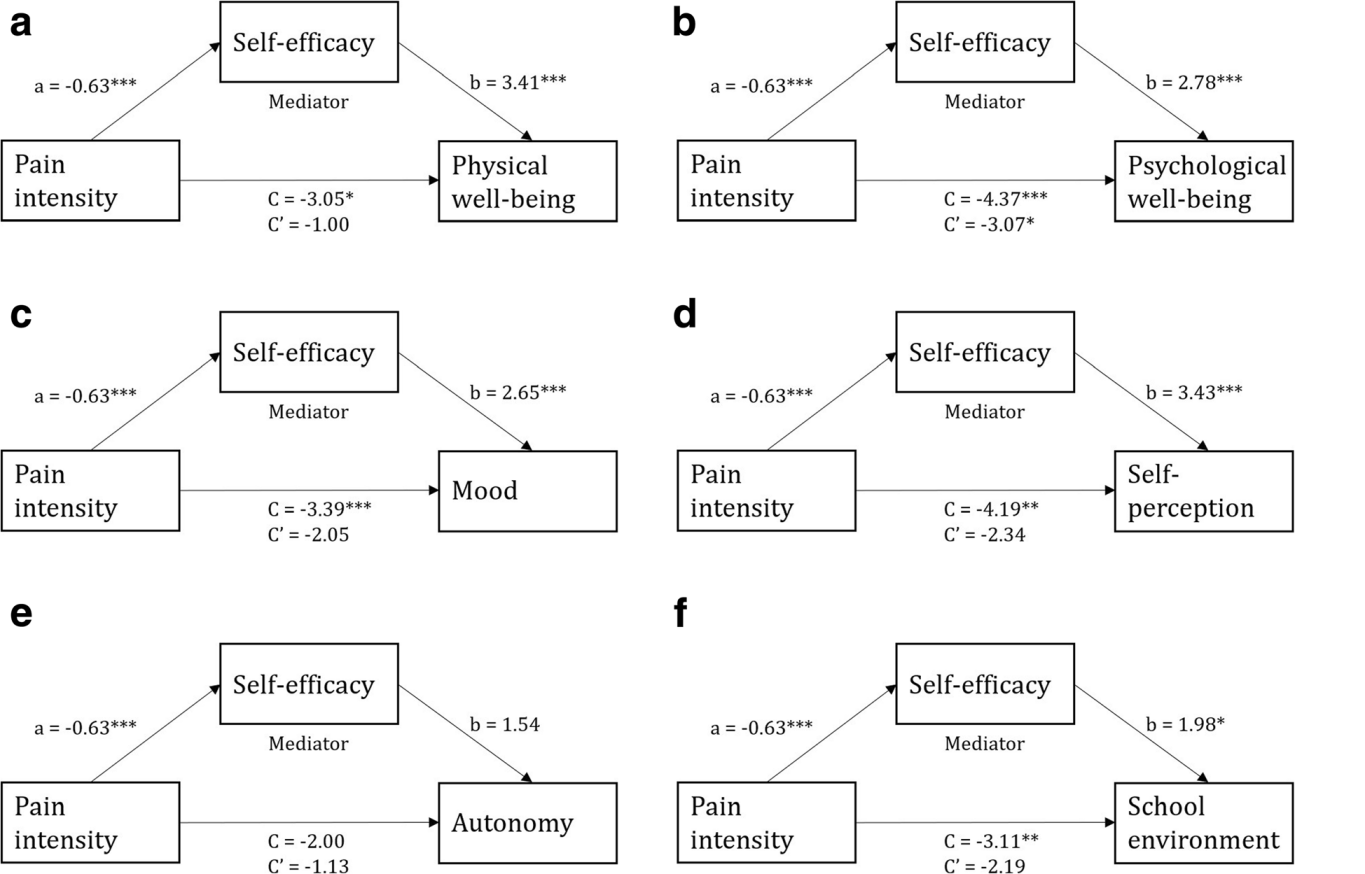
Mediation by self-efficacy of the association between pain intensity and the scores for HRQOL sub-scales **a** physical well-being, **b** psychological well-being, **c** mood, **d** self-perception, **e** autonomy and **f** school environment; *p* < 0.05*, *p* < 0.01** and *p* < 0.001***. Path a and b depict the indirect effects through the mediator. Path C represents the total effect and C' the direct path

The mediation effect was performed using the PROCESS macro developed by Hayes [41], herein we controlled for SES (entered as a covariate). A significant indirect effect was found for the HRQOL sub-scales: physical well-being (B = − 2.05; 95% CI [− 3.64 to − 0.56]), psychological well-being (B = − 1.30; 95% CI [− 2.96 to − 0.20]), mood (B = − 1.34; 95% CI [− 3.08 to − 0.19]), self-perception (B = − 1.85; 95% CI [− 3.65 to − 0.50]), autonomy (B = − 0.87; 95% CI [− 2.12 to − 0.03]) and school environment (B = − 0.92; 95% CI [− 2.73 to − 0.01]). Non-standardized estimates of the Bs of the associated variables are shown in Fig. 2. The direct paths (C′) between pain intensity and physical well-being, mood and school environment were no longer significant, which indicated that these associations were completely mediated by self-efficacy.

Approximately half of the reductions in HRQOL sub-scale scores for physical well-being, psychological well-being, mood, self-perception, autonomy and school environment was explained by the mediating variable (indirect effect). Physical well-being had the highest indirect effect (67.2%) among the HRQOL sub-scales (Table 5). The calculation of direct and indirect effect as percentages was not was not applicable for the HRQOL sub-scale bullying due to opposite directions of these effects.

**Table 5:** Reduction in HRQOL sub-scales explained by the direct (pain intensity) and indirect (self-efficacy) effects presented as percentage (%)

HRQOL sub-scales:	Direct effect (%)	Indirect effect (%)
Physical well-being	32.8	67.2*
Psychological well-being	70.3	29.7*
Mood	60.5	39.5*
Self-perception	55.8	44.2*
Autonomy	56.5	43.5*
Relationship with parents	74.6	25.4
Financial relationship	86.2	13.8
Social support	85.7	14.3
School environment	70.4	29.6*
Bullying	–	–

*p* < 0.05*

## Discussion

This study described the pain experience (intensity, frequency, duration and location) of adolescents with persistent pain, assessed the association between pain intensity, GSE and HRQOL, and tested self-efficacy as a possible mediator of pain. Our findings demonstrated that the participants were affected by the intensity, duration, frequency and locations of their experienced pain. Pain intensity was associated with impairments in the scores for several sub-scales of HRQOL and GSE. Further, GSE was a significant mediator between pain intensity and the HRQOL sub-scales of physical well-being, psychological well-being, mood, self-perception, autonomy and school environment. Up to 67% of the reduction in these respective HRQOL sub-scales was explained by the mediating variable (indirect effect).

Considering that the study sample was recruited from a school-based setting, and that headaches were the most commonly reported pain (88.5%), the overall presence of pain could be categorized as severe, with a mean pain intensity score of 5.4 (VAS) [52]. However, epidemiological studies have reported similar mean pain intensity scores ranging from 4.5 to 5.6 [2, 8]. Our data also revealed several gender differences: girls reported higher scores for pain intensity (VAS 5.7) compared with boys (VAS 4.2). Although all participants experienced persistent multi-site pain, girls reported pain in a greater number of body regions. These findings are consistent with the literature showing that headache is the most commonly reported type of pain, and that girls in late adolescence seem to experience more intense and frequent pain of longer duration than that experienced by boys, and more often have pain in multiple sites [7, 9, 11, 27]. Because pain is known to impact HRQOL, our findings predictably identified a gender difference in HRQOL sub-scale scores, with generally higher scores for boys than for girls. Higher HRQOL in adolescence in boys compared with girls is consistent with previous reports [53–56]. Data from 12 European countries (*n* = 21,590) showed no gender difference in HRQOL of young children; however, with increasing age, HRQOL in girls declined significantly compared with that in boys [56]. However, given that our study population was considered homogenous with respect to age, we were not able to perform any statistical inference concerning age.

Our findings revealed that pain intensity was negatively associated with all sub-scales of HRQOL and GSE, and that the participants generally reported low scores for HRQOL. However, in our regression analyses of pain intensity (independent) and sub-scales of HRQOL (dependent), the non-standardized estimates of B explained the difference in HRQOL in our study with that reported in an earlier published study, which used 10 sub-scales of KIDSCREEN-52 in a school-based population of children and adolescents (*n* = 1099) [27]. In this earlier school survey, the most impaired sub-scales of HRQOL for adolescents with persistent pain were psychological well-being, mood, self-perception, autonomy and school environment; this was generally consistent with our findings. However, unlike the earlier study, we did not identify any significant relationship between pain intensity and the HRQOL sub-scale autonomy, while our data showed a significant relationship between pain intensity and the scores for the HRQOL sub-scales of physical well-being and social support. These findings may relate to those of previous studies, which showed that persistent pain may result in periods of isolation from peers and, thus, absence from school, everyday physical activities and other social activities [3, 15]. Adolescents have reported that one of the most important things for their quality of life is to be social together with friends [57], and children and adolescents with persistent pain are commonly reported to have reduced social functioning and reduced physical activity levels [9, 58–60].

We hypothesized that self-efficacy would play a role as a possible mediator between pain and HRQOL. Interestingly, self-efficacy, a well-known approach to evaluating effects on a person’s cognition, did not only mediate the relationship between pain intensity and scores for HRQOL sub-scales connected with the adolescent’s perception of themselves, such as psychological well-being, mood and self-perception, but we showed that a reduction in self-efficacy also appeared to play a role in other HRQOL sub-scales, such as school environment. These findings are consistent with previous research that has shown that higher scores for self-efficacy in adolescents with chronic pain were associated with improved school functioning and lower school-related disability [35, 36]. Further, earlier studies showed that higher self-efficacy positively influences academic achievement and the likelihood of remaining in school [61]. Moreover, the highest indirect effect was found for the HRQOL sub-scale physical well-being, which is an important finding given that a reduction in physical well-being in adolescence is an indication of an impaired physical activity level, which is considered as a key component of a healthy lifestyle, herein self-efficacy is identified as a determinant for physical activity [62, 63]. A systemic review with meta-analyses by Ashford and colleagues discussed numerous ways to change self-efficacy, and reported that interventions, including feedback on past performance, feedback on performance compared with others and vicarious experience (role model), produced the highest levels of self-efficacy [64]. Bandura [65, 66] defined the concept of self-efficacy as a self-regulatory mechanism by which it is possible to change as a result of being motivated by others or through goal-setting and education. Thus, enhancing self-efficacy seems to be an important intervention strategy when aiming to improve HRQOL in adolescents with persistent pain.

### Strengths and limitations

All data analysed were cross-sectional, so no causal relationships could be identified. We could not test statistically the possible effect of gender due to the limited sample size and the homogeneity of the sample (a great majority were girls). Moreover, we were not able to control for other possible confounders as medication use. Hence, larger samples are recommended in future studies. The mediation model seeks to identify underlying mechanisms between observed associations but is of exploratory nature. Thus, this current meditation model is based on our assumptions and understanding of this research area, e.g. we can only assume causality and direction of the direct and indirect effect. Our findings are exploratory and should be verified and replicated in future and large studies and may only be generalized to a school-based population of adolescents with persistent and weekly pain. The effects may be over-estimated due to the shared source of variance. However, we consider that our findings shed new light on the underlying mechanisms of the association between pain and HRQOL in a sample from a school-based population of adolescents. We do not have any data regarding the 34 individuals who initially enrolled but were lost after registration; thus, the recruited adolescents might be those who were most interested because they had more severe pain. Hence, the findings may not be generalizable to the general population. A strength of the study is that we used well-validated questionnaires; however, the instrument for self-reported pain measures (LPQ) had a 3-month recall period for pain location, which might be a long period for adolescents to remember and may have reduced the validity of the data. In contrast, KIDSCREEN-52 used a 1-week recall period, which has been shown to be advantageous [16, 67].

### Clinical implications

Our findings provide new insight by showing that the association between pain intensity and HRQOL in a school-based sample of adolescents with persistent pain was explained by the mediating variable self-efficacy. Thus, this study extends previous assumptions and empirical research and shows that in future interventions for pain management, promoting self-efficacy could be beneficial for HRQOL. Given that research evidence has identified numerous ways to change self-efficacy [64–66], these findings may contribute to the design of more effective pain-management interventions that promote HRQOL in adolescents with persistent pain. Finally, regarding the adolescents’ school environment, teachers and health care nurses should be aware of targeting self-efficacy as a strategy to increase HRQOL.

## Conclusions

This study suggested that a school-based sample of adolescents with persistent pain had impaired HRQOL, which consequently affected all aspects of their everyday life and indicated the need for future targeted interventions. Our findings revealed that up to 67% of the reduction in the HRQOL sub-scale scores for physical well-being, psychological well-being, mood, self-perception, autonomy and school environment was explained by the mediating variable, self-efficacy. These data provide insight to the underlying mechanisms of the associations between pain and HRQOL in adolescents and have important implications for the future practice of pain management interventions, which should aim to increase HRQOL by promoting self-efficacy.

## Data Availability

The datasets used and/or analysed during the current study are available from the corresponding author on reasonable request.

## Abbreviations

GSE: General self-efficacy
HRQOL: Health-related quality of life
LPQ: Lübeck Pain-Screening Questionnaire
VAS: Visual Analog Scale
